# Reflections on intraoperative PTH monitoring and frozen biopsy in managing persistent hypercalcemia post-kidney transplant

**DOI:** 10.1097/JS9.0000000000001304

**Published:** 2024-03-05

**Authors:** Dan Shan

**Affiliations:** a Department of Biobehavioral Sciences, Columbia University, New York, USA; b Faculty of Health and Medicine, Lancaster University, Lancaster, UK

*Dear Editor*,

I wish to express my thoughts regarding the recent publication titled ‘Predictive factors for persistent hypercalcemia following parathyroidectomy in patients with persistent hyperparathyroidism after kidney transplantation: a retrospective cohort study’^1^. This study provided valuable insights into the underlying factors for continued hypercalcemia after hyperparathyroidism surgery in kidney transplant patients. The authors Kim *et al*. emphasized the necessity of confirming parathyroid lesions via frozen biopsy or intraoperative parathyroid hormone (Io-PTH) monitoring. This real-time guidance may help surgeons assess PTH level reductions and ensure the surgery’s extent is sufficient to avoid unnecessary parathyroid gland removal and achieve normokalemia after parathyroidectomy. However, a detailed discussion of these points was lacking in their initial publication. Io-PTH monitoring involves a complex process, and defining the so-called ‘appropriate surgery extent’ is challenging in clinical practice. Addressing these challenges requires careful consideration of several aspects.

Although prior research indicated that a substantial reduction in Io-PTH levels can guide the appropriate resection of parathyroid tissue and is associated with the successful elimination of parathyroid hyperfunction and a reduced risk of persistent hypercalcemia after surgery^2^, it remains unclear what degree of Io-PTH decrease relative to pre-excision levels should be considered effective and sufficient. While a decrease of at least 60–70% from pre-excision to 10–30 min post-excision was strongly linked with the resolution of hyperparathyroidism, the Miami criterion – a 50% reduction from the highest pre-excision PTH level at 10 min post-excision – did not seem to be a reliable indicator^3^. The appropriate timing of Io-PTH measurement is also elusive. A randomized trial found that extending Io-PTH measurement beyond 10 min did not improve outcomes and led to higher costs, utilizing a greater than 60% reduction in PTH levels from preoperative to 10 min post-excision as a benchmark to end the surgery^4^. This finding was corroborated by another study, which observed no substantial difference in Io-PTH levels between 10 and 20 min post-excision^2^. However, a study focusing on patients primarily undergoing total parathyroidectomy noted a marked decrease in Io-PTH levels between 10 and 20 min post-excision^5^.

Kim *et al*.^1^ found that a greater than 88% drop in preoperative PTH level by the first postoperative day acted as a protective factor against persistent hypercalcemia following parathyroidectomy. Given that they are now starting to monitor Io-PTH^1^, it would be prudent for them to reconsider the drop rate of Io-PTH relative to baselines across various time intervals. Ultimately, identifying an appropriate Io-PTH level drop before surgery completion could enhance surgical efficiency (i.e. avoid repetitive operative procedures), eliminating the need for a post-excision observation period to further assess surgical success, and potentially improving patient outcomes and experiences. For instance, conducting prospective studies to explore the relationship between decreasing Io-PTH levels at various time points (e.g. immediately post-removal, at 1, 5, and 10 min after removal) during surgery and the success of the procedure could be beneficial. Moreover, future research could consider analyzing large numbers of cases with big data and machine learning methods to identify patterns of Io-PTH decline that have predictive values.

In addition, although achieving normokalemia following parathyroidectomy should be a great concern, it should be borne in mind that the Io-PTH levels following gland removal do not predict the likelihood of postoperative hypoparathyroidism in kidney transplant recipients. Instances of severe hypoparathyroidism and hypocalcemia have occurred even when patients exhibit PTH levels more than twice the upper normal limit within 15–20 min post-excision^6^. In this context, the authors highlighted the value of employing frozen section biopsy during parathyroidectomy for on-the-spot histopathological examination of the excised tissue. This may ensure the precise identification and conservation of parathyroid glands. Such careful confirmation serves to avoid the accidental excision of non-abnormal parathyroid tissue, thus reducing the potential for post-surgical complications, including hypocalcemia.

Finally, while the frozen section technique was generally considered a reliable method for tissue identification during parathyroidectomy, it is susceptible to diagnostic inaccuracies stemming from artifacts related to the freezing process, errors in sampling, and inaccuracies in judgment^7^. Differentiating between lymphoid tissue and the parathyroid gland poses a particular challenge. It was advisable to conduct a touch-prep procedure prior to the frozen section when identifying the parathyroid gland. This rapid cytological technique has proven to be effective and reassuring in minimizing the likelihood of misidentification between the parathyroid gland and lymph nodes^7^.

It would be beneficial to learn from the authors about their considerations of these factors in their ongoing trials related to Io-PTH monitoring and frozen biopsy.

## Ethical approval

Not applicable.

## Consent

Not applicable.

## Sources of funding

Not applicable.

## Author contribution

Dan Shan contributed all.

## Conflicts of interest disclosure

I declare no conflicts of interest.

## Research registration unique identifying number (UIN)

Not applicable.

## Guarantor

Dan Shan.

## Data availability statement

Not applicable.

## Provenance and peer review

Not invited.
